# Response: Commentary: Analysis of SUMO1-conjugation at synapses

**DOI:** 10.3389/fncel.2018.00117

**Published:** 2018-05-01

**Authors:** James A. Daniel, Benjamin H. Cooper, Jorma J. Palvimo, Fu-Ping Zhang, Nils Brose, Marilyn Tirard

**Affiliations:** ^1^Department of Molecular Neurobiology, Max Planck Institute of Experimental Medicine, Göttingen, Germany; ^2^Institute of Biomedicine, University of Eastern Finland, Kuopio, Finland; ^3^Institute of Biomedicine, Research Centre for Integrative Physiology and Pharmacology, Turku Center for Disease Modeling, University of Turku, Turku, Finland

**Keywords:** SUMO1, SUMOylation, synapse, neuron, post-translational modification, antibodies, antibody specificity

Wilkinson et al. (2017) commented in this forum on a study of ours (Daniel et al., 2017), in which we report that the evidence for SUMO1-conjugation at synapses and of several synaptic proteins is equivocal. We present here—due to length restrictions—an abbreviated version of a response to Wilkinson et al. that appeared in the comments section of our original publication (Daniel et al., 2017).

## Validity of the His_6_-HA-SUMO1-KI model

Wilkinson et al. criticize our use of His_6_-HA-SUMO1-KI mice to study SUMO1-conjugation. We generated the His_6_-HA-SUMO1-KI to study SUMO1-conjugation of synaptic proteins, but our initial analyses of this model did not yield evidence for synaptic SUMO1-conjugation (Tirard et al., 2012). To address the discrepancy between our findings and previous reports, we performed our recent study (Daniel et al., 2017). Using immunoaffinity purification of proteins from wild-type (WT) and His_6_-HA-SUMO1-KI brain tissue, we tested eight candidate SUMO1-substrates for His_6_-HA-SUMO1-conjugation (the transcription factor Zbtb20 and the synaptic proteins synapsin-1A, gephyrin, GluK2, RIM1, syntaxin-1A, synaptotagmin-1, and mGluR7). Only Zbtb20, which we had previously identified in screens for SUMO1-conjugated brain proteins (Tirard et al., 2012), yielded evidence of SUMO1-conjugation in Western blot analyses. For the synaptic proteins, no bands with the appropriate SUMO1-conjugation-induced size shift were detected, challenging the notion that the tested proteins are *bona-fide* SUMO1-substrates (Daniel et al., 2017).

Wilkinson et al. highlight that overall SUMO1-conjugation is reduced by ~20–30% in the His_6_-HA-SUMO1-KI (Tirard et al., 2012; Daniel et al., 2017). Consequently, based on the same argumentation that some of the commentary-authors used before (Luo et al., 2013; Henley et al., 2014), they dismiss the validity of the His_6_-HA-SUMO1-KI as a SUMO1-conjugation reporter, and attribute the lack of synaptic SUMO1-conjugation in our studies to the reduction in SUMO1-conjugation levels and the presence of the His_6_-HA-tag.

We have consistently acknowledged the ~20–30% reduction of SUMO1-conjugation in His_6_-HA-SUMO1-KI brain and the consequent possibility that some SUMO1-conjugated proteins might be too transient, unstable, or rare to be detectable (Tirard et al., 2012; Daniel et al., 2017). However, many previously identified SUMO1-substrates were detected with the His_6_-HA-SUMO1-KI, along with novel SUMO1-substrates (Tirard et al., 2012), including Zbtb20, which was subsequently found in other proteomic screens for SUMOylation substrates (Becker et al., 2013; Hendriks et al., 2017) and validated in the present study (Daniel et al., 2017). We therefore regard it as unlikely that all seven synaptic candidate SUMO1-substrates we tested escaped our detection, e.g., due to a complete occlusion effect of the ~20–30% reduction in overall SUMO1-conjugation levels or the influence of the His_6_-HA-tag. In support of our *in vivo* data, Western blot analyses of SUMO1-conjugation of recombinant Zbtb20, synapsin-1, gephyrin, and GluK2 in fibroblasts that co-expressed HA-tagged SUMO1 showed that only Zbtb20 is SUMO1-conjugated (Daniel et al., 2017). Because the candidate proteins and HA-SUMO1 were strongly overexpressed in these experiments, we consider it unlikely that a lack of SUMO1-conjugation of the synaptic candidate proteins is due to an intrinsic ~20–30% decrease of HA-SUMO1-conjugation (as proposed by Wilkinson et al. for the His_6_-HA-SUMO1-KI). Furthermore, replacement of SUMOs by tagged variants—even with larger tags than His_6_-HA—is well-tolerated in the model organisms tested so far (Panse et al., 2004; Kaminsky et al., 2009; Miller et al., 2010).

## Use of wild-type material in our analyses

Wilkinson et al. state that “Most of the experiments reported by Daniel et al. use a knock-in (KI) mouse that expresses His_6_-HA-SUMO1 in place of endogenous SUMO1,” overlooking our analyses in WT mice. In fact, we immunopurified Zbtb20, synapsin-1, gephyrin, GluK2, RIM1, and syntaxin-1A from WT mouse brain and assessed the input and immunoisolated proteins by Western blotting using antibodies against the different proteins. Zbtb20 exhibited unequivocal evidence of protein species with molecular weight shifts that likely represent SUMO-conjugation. No candidate synaptic proteins exhibited an apparent molecular weight shift indicative of SUMO-conjugation in WT (or His_6_-HA-SUMO1-KI) samples (Daniel et al., 2017). Because these experiments did not allow us to determine whether SUMO-conjugation is due to SUMO1, SUMO2, or SUMO3, our data also address the criticism by Wilkinson et al. that we did not examine SUMO2/SUMO3-conjugation. Moreover, we did not focus on SUMO2/SUMO3 because most previous synaptic SUMOylation studies focused on SUMO1—except studies on mGluR7 (Choi et al., 2016) and gephyrin (Ghosh et al., 2016). Wilkinson et al. also mention that we did not use WT material in our analyses of candidate SUMO1-conjugated proteins in subcellular brain fractions, which we have now addressed (see below).

## Immunodetection of SUMO1

Wilkinson et al. cite publications reporting the presence of SUMO1, SUMO1-conjugated proteins, and/or components of the SUMOylation machinery in synapses as assessed by immunolabeling of cells and tissue or Western blot analyses of subcellular brain fractions. These studies involved anti-SUMO1 antibodies that Wilkinson et al. refer to as “validated.” However, these anti-SUMO1 antibodies were never validated by using SUMO1-KO samples as negative control. This is a major omission, particularly given the many different fixation, permeabilization, and staining protocols used.

Large-scale studies indicate that only ~50% of commercially available antibodies can be used to reliably assess protein distribution in tissue (Baker, 2015). Correspondingly, we show (i) that “synaptic” signals generated with a “validated” anti-SUMO1 antibody in cultured WT neurons show no significant difference to SUMO1-KO samples, and (ii) that most of the anti-SUMO1-positive bands in synaptic fractions of mouse brain are equally evident in His_6_-HA-SUMO1-KI and SUMO1-KO samples (Zhang et al., 2008; Daniel et al., 2017). These data indicate that the anti-SUMO1 antibody generates non-specific signals that can be erroneously interpreted as synaptic SUMO1-conjugation. This conclusion is supported by the fact that anti-SUMO1 immunolabeling in neuronal dendrites is punctate in some studies (Martin et al., 2007; Konopacki et al., 2011; Loriol et al., 2013; Craig et al., 2015) and relatively homogeneous in others (Kantamneni et al., 2011; Craig et al., 2012; Ghosh et al., 2016). We note that Wilkinson et al. cite a paper by Hasegawa et al. (2014) in the context of evidence for synaptic localization of SUMO1-conjugates. The authors demonstrate that SUMO1, SUMO2, and SUMO3 immunoreactivity is present in the nuclei of many cell types throughout the brain (Hasegawa et al., 2014), but did not employ antibodies against synaptic markers and did not make claims about SUMO-immunoreactivity at synapses.

Wilkinson et al. mention the low SUMO1 signal intensity in nuclei of WT and His_6_-HA-SUMO1-KI neurons. We acknowledge that the nuclear anti-SUMO1 immunolabeling is 20–30% higher in WT neurons than in His_6_-HA-SUMO1-KI neurons, as we previously noted (Tirard et al., 2012; Daniel et al., 2017). In some previous studies (e.g., Gwizdek et al., 2013; Jaafari et al., 2013) images of anti-SUMO1 immunolabeling are saturated, which we wanted to avoid. In our experiments, we made a considerable effort to recapitulate the methods of immunolabeling of previous studies to eliminate a methodological basis for the differences between our observations and previous studies. Due to the large number of images acquired per neuron we also used minimal laser power to prevent bleaching.

**Figure 1:**
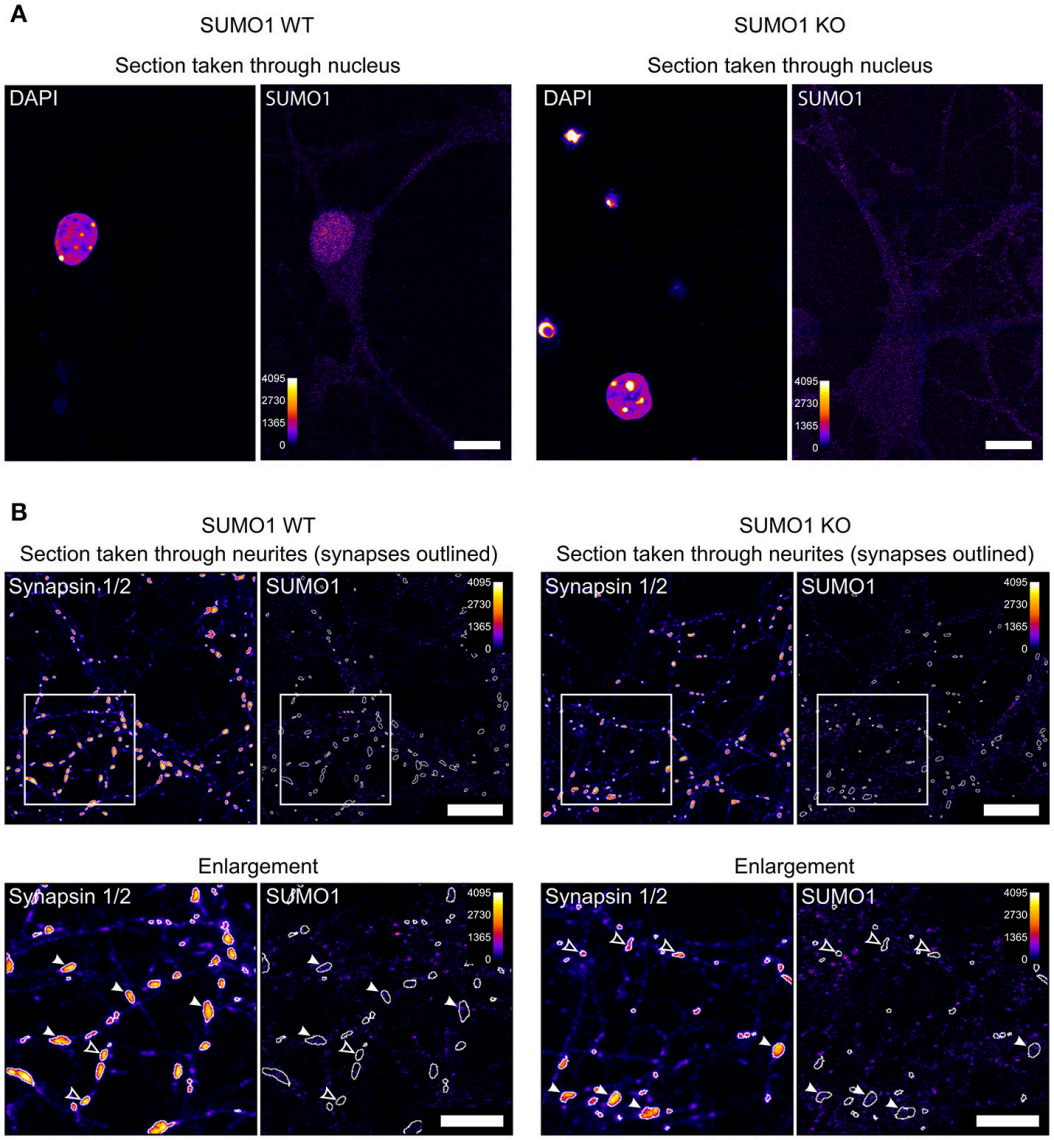
Anti-SUMO1 immunolabeling in nuclei and neurites of WT and SUMO1-KO neurons. **(A)** Hippocampal neurons were fixed, permeabilized using Triton X-100, immunolabeled with anti-SUMO1 antibodies, and imaged (Daniel et al., 2017). The two left images show a representative confocal section through the neuronal nucleus/soma of a WT neuron, labeled with DAPI, and anti-SUMO1 antibodies. The right images show a section through a SUMO1-KO neuron. The Fiji fire heat map lookup table was applied to images to visualize fluorescence intensity (0–4,095, as shown in scale). The WT neuron shows nuclear anti-SUMO1 immunolabeling, which is absent in the SUMO1-KO neuron. These images were taken from the same dataset that was used to generate Figure 16 in Daniel et al. (2017). Scale bars, 10 μm. **(B)** Hippocampal neurons were fixed, permeabilized using digitonin, immunolabeled with anti-SUMO1/anti-synapsin antibodies, and imaged (Daniel et al., 2017). The left images show a representative confocal section through the neurites/synapses of a WT neuron, labeled with anti-synapsin and anti-SUMO1 antibodies. The right images show a section through a SUMO1-KO neuron. The images in the lower panels show the detail of an inset region (400 × 400 pixels) from the upper panels. The fluorescence intensity of the images is represented using the fire LUT from Fiji. The Fiji fire heat map lookup table was applied to images to visualize fluorescence intensity (0–4,095, as shown in scale). White-outlined regions of interest (ROIs) around synapsin-positive puncta were generated by a custom Fiji macro and are shown applied to anti-SUMO1 images as well. Synapsin puncta in which SUMO1 signal is visible are marked with white arrowheads in both WT and SUMO1-KO cultures. Synapsin puncta in which SUMO1 signal was essentially undetectable are marked with open arrowheads in both WT and SUMO1-KO cultures. These images were taken from the same dataset that was used to generate Figure 12 in Daniel et al. (2017). Scale bars, 10 μm.

For illustrative purposes, we provide unprocessed images from the datasets used in our study (Figures 1A,B). Nuclear immunolabeling in Triton-X-100-permeabilized neurons from WT mice is much stronger than in the surrounding cytoplasm and neurites (Figure 1A). In SUMO1-KO neurons, the nuclear labeling is not visible. This is reflected by quantitative analyses in our original study and validates the conclusion that the SUMO1-KO results in a dramatic loss of specific anti-SUMO1 immunolabeling in nuclei (Daniel et al., 2017). The relatively weak extranuclear anti-SUMO1 immunolabeling in Triton-X-100-permeabilized neurons that we observed was also noted by the Henley-group and led to their use of digitonin as a permeabilization agent for SUMO1 immunolabeling in some of their studies. The relatively weak anti-SUMO1 immunolabeling in neurons also highlights an advantage of the His_6_-HA-SUMO1-KI model, given the strong and specific immunolabeling achieved with anti-HA in this system.

Unprocessed sample images of neurites of digitonin-permeabilized neurons are also provided (Figure 1B), generated by our Fiji macro used for synaptic anti-SUMO1 intensity quantification (Daniel et al., 2017). Anti-SUMO1 immunolabeling in neurites is punctate, but most anti-SUMO1 puncta do not correspond to synapsin-positive structures, and puncta are equally evident in WT and SUMO1-KO neurons (Figure 1B). These images reflect our quantitation of these data in the original study (Daniel et al., 2017). Regarding the notion of Wilkinson et al. that the “low detection levels would almost certainly rule out visualization” of synaptic SUMO1, we note that both visual (qualitative) examination and quantification of “synaptic” anti-SUMO1 labeling show that anti-SUMO1 immunolabeling at synapses is not different between WT and SUMO1-KO neurons. Presumably, if anti-SUMO1 immunolabeling at synapses were specific but relatively weak, the anti-SUMO1 immunolabeling should still be higher in WT than in SUMO1-KO neurons. Thus, specific anti-SUMO1 immunolabeling is either absent or of such low abundance as to be undetectable using our methods. In agreement with our findings, a recent study also shows virtually no overlap between anti-SUMO1 and anti-synaptophysin immunolabeling in brains of mice that overexpress SUMO1 (Matsuzaki et al., 2015).

Regarding our Western blot analyses of brain subcellular fractions, Wilkinson et al. argue that we should have used WT mice and not His_6_-HA-SUMO1-KI mice to compare to the SUMO1-KO samples, presumably because WT mice have a ~20–30% higher overall SUMO1-conjugation level than His_6_-HA-SUMO1-KIs. What Wilkinson et al. do not acknowledge is that our analysis shows that anti-SUMO1 signals in synapses of cultured neurons and in synaptic subcellular fractions are equally well detected in SUMO1-KO samples, indicating that they are of a non-specific nature (Daniel et al., 2017). Nevertheless, we generated new subcellular fractions from WT and SUMO1-KO brains, and assessed them by Western blotting with six different anti-SUMO1 antibodies. The corresponding data (Supplementary Figure 1) show that (i) anti-SUMO1 antibodies exhibit non-specific cross-reactivity with proteins in SUMO1-KO samples, (ii) there is little correspondence between datasets obtained with the different antibodies, and (iii) the vast majority of protein bands that are detectable in WT synaptic fractions by these anti-SUMO1 antibodies are equally detectable in synaptic SUMO1-KO fractions. These observations stress the requirement of KO controls and demonstrate that the evidence for SUMO1-conjugated proteins in synaptic fractions is equivocal.

## Functional studies

Wilkinson et al. state that the SUMOylation of many synaptic proteins has been functionally “validated,” and criticize that our experiments were confined to immunolabeling and Western blotting analyses. We did not examine the functional consequences of SUMO1-conjugation of candidate synaptic proteins because we did not obtain evidence that these proteins were SUMO1-conjugated. Furthermore, we felt that the standard functional analyses of SUMO1-conjugation are not really helpful for the resolution of the current controversy. In general, some proposed function of the WT form of a given protein is typically compared to a variant in which a proposed SUMO-conjugated lysine residue is mutated to abolish SUMO-conjugation. Under optimal conditions, this functional comparison is conducted on a background where the expression of the endogenous protein is blocked. However, it is impossible to be certain that functional consequences of the lysine mutation are specifically due to the blockade of SUMOylation because the mutation might have SUMOylation-independent consequences, e.g., by affecting protein structure, interactions, or other post-translational modifications. This problem is aggravated when WT and lysine-mutant variants of proteins are overexpressed and/or compared in a WT background. Thus, in cases where the *in vivo* SUMOylation of a given protein and the identity of a given SUMOylation site are equivocal, the functional consequences of a corresponding lysine mutation must be interpreted carefully.

## Anti-Gluk2 antibodies

Wilkinson et al. allege that a key flaw in our analyses of GluK2 SUMOylation is “that the C-terminal anti-GluK2 monoclonal rabbit antibody used does not recognize SUMOylated GluK2 because its epitope is masked by SUMO conjugation.”

While this might be a relevant point, we did not see it raised or systematically addressed in any paper. Further, it is difficult to deduce why the antibody we used (i.e., MerckMillipore rabbit monoclonal anti-GluK2 antibody NL9; rmAb-MerckMillipore-NL9) might not recognize SUMOylated GluK2. We assume, based on the time of publication and corresponding information in the corresponding methods text, that the anti-GluK2 antibody used in the first study to detect SUMO1-conjugated GluK2 (Martin et al., 2007) was Upstate rabbit polyclonal anti-GluK2 antibody 06/309 (rpAb-Upstate-06/309). This antibody, which has been discontinued, was raised against a lysine-linked peptide representing the C-terminal 15 amino acids residues of rat GluK2 (Lys-HTFNDRRLPGKETMA). rmAb-MerckMillipore-NL9 was raised against the exact same sequence of the C-terminus of rat GluK2 (linked to keyhole limpet hemocyanin, KLH) as rpAb-Upstate-06/309 (i.e., KLH-HTFNDRRLPGKETMA). Given that the two antibodies relevant in this controversy were raised against exactly the same C-terminal GluK2-sequence, which is proximal to but does not include the proposed SUMO1-conjugation site K886, it is not apparent why SDS-denatured, SUMO1-conjugated GluK2 should be readily detectable on Western blots by rpAb-Upstate-06/309 but not by rmAb-MerckMillipore-NL9.

Further complicating the issue, two of the studies cited by Wilkinson et al. actually employed rmAb-MerckMillipore-NL9 to detect SUMO-conjugated GluK2 (Konopacki et al., 2011; Zhu et al., 2012). Konopacki et al. (2011) state under “Materials and Methods” in the “Supporting Information” part of their publication that rmAb-MerckMillipore-NL9 was used to detect purified GluK2 C-termini in *in vitro* assays, and show Western blots of apparently *in-vitro*-SUMO1-conjugated C-terminal fragments of GluK2. According to the “Materials and Methods” part, Zhu et al. (2012) used rmAb-MerckMillipore-NL9 for all analyses of SUMO-conjugation of GluK2. Ignoring, for the sake of the argument, other issues with the study by Zhu et al. (2012) and taking the data provided at face value, one has to assume again that rmAb-MerckMillipore-NL9 can detect SUMO1-conjugated GluK2.

Finally, we performed new Western blot analyses with two additional anti-GluK2 antibodies, Abcam rabbit polyclonal anti-GluK2 antibody 66440 (rpAb-Abcam-66440), which was raised against an N-terminal epitope, and Alomone rabbit polyclonal anti-GluK2 antibody AGC-009 (rpAb-Alomone-AGC-009), which was raised against a C-terminal epitope (amino acid residues 858–870) that excludes the proposed GluK2 SUMOylation site. The corresponding Western blots show no evidence of a GluK2-positive band with shifted molecular weight, supporting our conclusion (Daniel et al., 2017) that evidence for GluK2 SUMOylation remains equivocal. Representative Western blots are shown in the comments section of our original study (Daniel et al., 2017).

## Conclusion

We maintain that the His_6_-HA-SUMO1-KI mouse line is a reliable and useful tool for the localization and identification of SUMO1-substrates, particularly when used alongside WT and SUMO1-KO mice, and further contend that the role of SUMO1-modifications in the function of synaptic proteins and synapses remains—at least—unclear. We published our study (Daniel et al., 2017) to highlight discrepancies in the published record and to encourage activities toward a consensus set of criteria based on which SUMO-conjugation of a candidate protein can be verified in neurons *in vivo*. Using landmark studies in other areas of SUMO biology for guidance, we proposed such a set of criteria in our study (Daniel et al., 2017). We expect that adherence to these criteria, along with the development of genetically engineered mice that allow the unequivocal mass spectrometric identification of SUMO-conjugated peptides in proteolytic digests of proteins from mouse brain, subcellular brain fractions, or purified protein fractions, will ultimately resolve the present controversy.

## Ethics statement

All animal experiments were performed in accordance with the guidelines for the welfare of experimental animals issued by the State Government of Lower Saxony (33.9-42502-04-13/1359), Germany, in compliance with European and NIH guidelines.

## Author contributions

JD, MT, and NB: wrote the first draft of the manuscript; JD, MT, and BC: prepared the figures. All authors then contributed ideas and writing to create the final version of the manuscript.

### Conflict of interest statement

The authors declare that the research was conducted in the absence of any commercial or financial relationships that could be construed as a potential conflict of interest.

## Supplementary material

Supplementary Figure 1 SUMO1-conjugated proteins in subcellular brain fractions. Brains from adult WT and SUMO1-KO mice were subjected to subcellular fractionation as detailed in Daniel et al. (2017). Western blot analyses of the fractions using anti-GluN1 and anti-synaptophysin antibodies validate the fractionation procedure. Western blot analyses of the fractions using six different anti-SUMO1 antibodies confirm the strong enrichment of SUMO1 candidates in nuclear fractions (P1) but not in synaptic fractions (LP1, SPM). H, homogenate; P1, nuclear pellet; S1, supernatant after P1 sedimentation; P2, crude synaptosomal pellet; S2, supernatant after P2 sedimentation; P3, cellular membrane, and organelle fraction; S3, supernatant after P3 sedimentation; LP1, lysed synaptosomal membranes; LS1, supernatant after LP1 sedimentation; LP2, crude synaptic vesicles; SPM, partially purified synaptic plasma membranes. Arrows indicate free SUMO1; stars indicate non-specific bands detected by the anti-SUMO1 antibodies.
