# Supplementary figures and images for: Response: Commentary: Analysis of SUMO1-conjugation at synapses
Source: Front Cell Neurosci. 2018 May 1;12:117. doi: 10.3389/fncel.2018.00117 (PMC5938361; doi:10.3389/fncel.2018.00117)

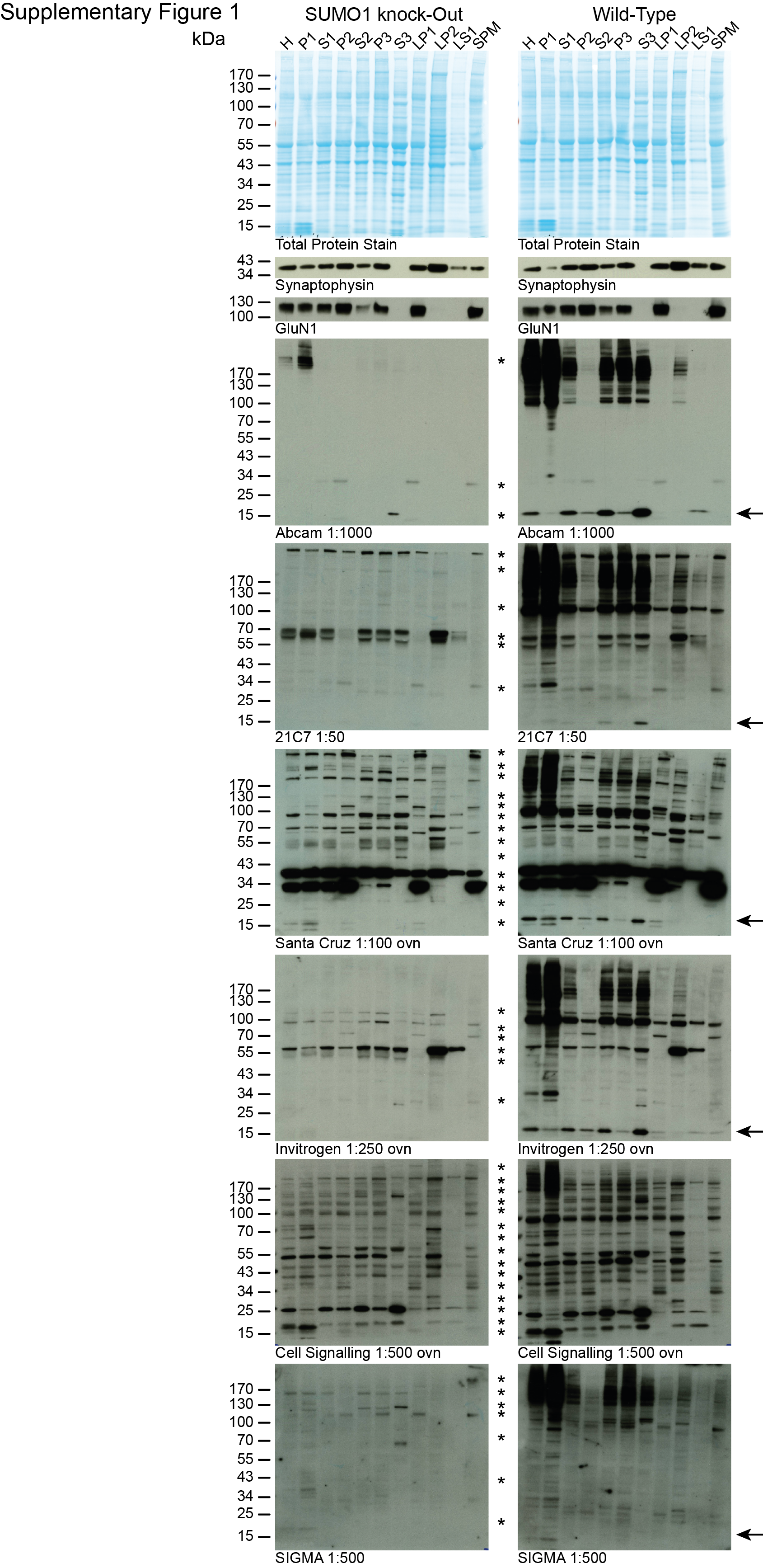

Supplement: Supplementary Figure 1 — SUMO1-conjugated proteins in subcellular brain fractions. Brains from adult WT and SUMO1-KO mice were subjected to subcellular fractionation as detailed in Daniel et al. (2017). Western blot analyses of the fractions using anti-GluN1 and anti-synaptophysin antibodies validate the fractionation procedure. Western blot analyses of the fractions using six different anti-SUMO1 antibodies confirm the strong enrichment of SUMO1 candidates in nuclear fractions (P1) but not in synaptic fractions (LP1, SPM). H, homogenate; P1, nuclear pellet; S1, supernatant after P1 sedimentation; P2, crude synaptosomal pellet; S2, supernatant after P2 sedimentation; P3, cellular membrane, and organelle fraction; S3, supernatant after P3 sedimentation; LP1, lysed synaptosomal membranes; LS1, supernatant after LP1 sedimentation; LP2, crude synaptic vesicles; SPM, partially purified synaptic plasma membranes. Arrows indicate free SUMO1; stars indicate non-specific bands detected by the anti-SUMO1 antibodies. [file Image_1.png]
